# 

**Published:** 1920-05-01

**Authors:** 


### Gifts and Bequests.

Mr. Jesse Hind, of Edwalton, has left £200 to the Nottingham General Hospital.

Mr. D. H. Robson-Burrows, of Bournemouth, has left the residue of his estate, which will exceed £10,000, to the Royal Victoria and West Hants Hospital.

Mr. Thomas Arkell, of the Kingsdown Brewery, has left £500 to the Swindon Victoria Hospital and £100 each to Cirencester Hospital, Faringdon Hospital, and Fairford Hospital.

Mr. George Barbour, of Bolesworth Castle, Cheshire, left estate to the gross value of £1,311,253. Amongst other legacies the testator left £5,000 to the Chester Royal Infirmary.

The Royal Berkshire Hospital has been left £500 under the will of the late Mr. R. J. Allsop.

A sum of £1,000 has been realised by Viscountess Sandhurst's bazaar at St. Bartholomew's Hospital on behalf of the Appeal Fund.

The Great Northern Central Hospital, Holloway, has received £80 from Major E. T. Kingscote, St. James's Palace (proceeds of concert), £52 10s. from Anglo-Egyptian Bank, £20 from Mr. J. T. Malcolm (Persia), £6 9s. from the staff of Messrs. Jones Bros. (per Miss Eva Johnson), proceeds of weekly collections in aid of League of Roses, £2 10s. from Mr. J. Hinds (Hawaii), and £804 from the Hospital Saturday Fund.

### Hospital Poultry-Keeping.

Following the partial solution suggested from time to time in these columns of the utilisation of "scraps," the Hendon Cottage Hospital is about to keep poultry, and the matron is appealing for some twenty pullets with which to make a start. It is hoped that the food bill will not be too heavy, because of the amount of "waste" stuffs available even in the most carefully ordered institution.

### NURSE REGISTRATION ACT, 1919.

### GENERAL NURSING COUNCIL.

The following persons have been appointed to form the first General Nursing Council under the Nurse Registration Act of 1919. The duty of the Council is to form and keep a register of nurses in accordance with the provisions of the Act:—

*Appointed by the Privy Council.*—Lady Hobhouse, Mr. J. C. Priestley, K.C.

*Appointed by the Board of Education.*—Hon. Mrs. Eustace Hills, Miss Batty Tuke, Bedford College.

*Appointed by the Minister of Health.*—The Rev. G. B. Cronshaw, Radcliffe Infirmary, Oxford; Dr. E. W. Goodall, M.D.; Dr. A. Bostock Hill, M.D.; Dr. Bedford Pierce, M.D.; Sir T. Jenner Verrall, M.D.

*Nurses Appointed by the Minister of Health.*—Miss A. Cattell, private practice; Mr. T. Christian, nurse, Banstead Lunatic Asylum; Miss A. Coulton, matron, Shadwell Children's Hospital; Miss R. Cox-Davies, R.R.C., matron, Royal Free Hospital; Miss A. Dowbiggin, C.B.E., R.R.C., matron, Edmonton Poor-Law Infirmary; Mrs. E. G. B. Fenwick, formerly matron St. Bartholomew's Hospital; Miss A. Lloyd-Still, C.B.E., R.R.C., matron, St. Thomas's Hospital; Miss M. MacCullum, Professional Union of Trained Nurses; Miss I. Macdonald, Royal British Nurses' Association; Miss A. M. Peterkin, General Superintendent, Queen Victoria Jubilee Institute for Nurses; Miss E. Smith, Welsh Superintendent, Queen Victoria Jubilee Institute for Nurses; Miss M. E. Sparshott, C.B.E., R.R.C., matron, Royal Infirmary, Manchester; Miss E. C. Swiss, health visitor for Willesden; Miss S. E. Villiers, matron, Stockwell Fever Hospital; Miss C. Worsley, matron, Liverpool Children's Hospital; Miss C. S. Yapp, matron, Ashton-under-Lyne Poor-Law Infirmary.

Mr. Priestley has been appointed to be the Chairman of the Council.

### The Book Market.

Chas. Griffin and Co., Ltd.: "Medical Handbook for the Use of Practitioners and Students." By R. S. Aitchison, M.D. 10s. 6d.

A. and C. Black, Ltd.: "Medieval Medicine." By James J. Walsh, K.C.M.G., M.D., etc. 7s. 6d.

John Murray: "An Index of Symptoms." By Ralph Winnington Leftwich, M.D. Seventh Edition Revised. By H. N. Warner Collins, B.Sc., M.R.C.S., L.R.C.P. 15s. net.

Oxford University Press: "The Nutrition of the Fœtus." By J. Morris Slemons. 3s. 6d. net.

Butterworth and Co. (India), Ltd., Calcutta: "Personal Hygiene." By W. R. Saney, M.D. Rs. 3 net.

John Lane: "Ward Tales." By E. Chivers Davies. 5s. net.

A. C. Fifield: "Diversions in Sicily." By H. Festing Jones. 6s. net. "Castellinaria and Other Sicilian Diversions." By H. Festing Jones. 6s. net.

Humphrey Milford, Oxford University Press: "Of the Imitation of Christ." "Edith Cavell" Edition. By Thomas à Kempis. 2s. 6d. net.

H. K. Lewis and Co., Ltd.: "The Link Between the Practitioner and the Laboratory." By C. Fletcher, M.B., B.S.Lond., and H. McLean, D.P.H., M.R.C.S., L.R.C.P. 4s. 6d. net.

### Matrons' and Sisters' Appointments.

Township Infirmary, Beckett Street, Leeds.—The following have been appointed ward sisters: Miss Catherine Jackson, trained at Manchester Royal Infirmary, has been sister at St. George's Military Hospital, Stockport, and has done private nursing; Miss Ivy Ellen Sarsby, trained at Leicester Royal Infirmary, where she was later ward sister; she has done private nursing at San Remo, and military and district nursing. Miss Eliza Jennings, trained at Hartlepool Hospital, where she was later sister; she has also been sister at Northumberland War Hospital. Miss Margaret Burchall, trained at Highfield Infirmary, Liverpool, where she was later staff nurse and ward sister. Miss Marie G. Baker, trained at Brownlow Hill Infirmary, Liverpool, and has since been sister-in-charge and matron under the British Red Cross Society at Denbigh and Buxton, and has done private nursing. Miss Mary Pollock, trained at Township Infirmary, Leeds, where she has since been staff nurse and charge nurse; she holds a certificate for invalid cookery and for the Central Midwives Board. Miss Elizabeth Macdonald has been appointed junior night superintendent; she was trained at Falkirk County Hospital and at Township Infirmary, Leeds, and holds certificates for invalid cookery and fever nursing and that of the Central Midwives Board.

Maternity Hospital, York.—Miss I. Cooper has been appointed sister. She was trained at Prescot Infirmary and Queen Charlotte's Hospital, at which latter institution she remained on the staff for one year. Miss Cooper has also been sister at Willesden Hospital and at Walsall General Hospital.

Selly Oak Hospital, Birmingham.—Miss L. V. Eaton has been appointed night sister. She was trained at Birkenhead Infirmary, and is at present assistant matron at the Mariners' Home, Egremont.

Evelina Hospital for Children, Southwark, S.E.—Miss M. Irene Lindars, the assistant matron, has been appointed matron, and Miss Ethel Emly, the senior of the sisters, has been promoted to be assistant matron. Miss Lindars received her children's training at the "Evelina," where she remained for four years; she then proceeded to Westminster Hospital for her general training, and afterwards returned to the "Evelina" as night sister, surgical sister, and assistant matron successively. Miss Emly took her children's training at the East London Hospital for Children, Shadwell, and later went to Addenbrooke's Hospital, Cambridge, for her general training; she then became sister-in-charge of an Auxiliary Hospital in Ayrshire, and later night sister at the "Evelina," being transferred after six months to be out-patient sister.

### The College of Nursing (Limited by Guarantee).

### EDINBURGH CENTRE.

A meeting of members was held at 8 Drumsheugh Gardens on Wednesday, April 21, for the purpose of considering a communication from the Council concerning the "Hours of Employment Bill as Affecting Nurses." Miss Cumming, R.R.C., presided. The discussion, in which private nurses took a leading part, showed that there was a unanimous feeling as to the wisdom of shortening hours, but some anxiety as to the rigidity of Government control. The findings of the meeting were forwarded to London for the consideration of the Council.

### BIRMINGHAM CENTRE.

On April 27 Dr. Thomas Wilson gave an interesting lecture on Gynæcology to an appreciative audience in the Lecture Theatre of the General Hospital. His graphic description of the difficulties which beset both the gynæcologist and the midwife practising amongst the poorer classes in a large industrial city was much appreciated.

